# Associations of Thrombocytopenia and Subthreshold Platelet Counts with Clinical Outcomes in Intensive Care Patients: A Prospective Cohort Study

**DOI:** 10.3390/jcm15207869

**Published:** 2026-10-12

**Authors:** Kamil Deveci, Maruf Boran, Kübra Elif Bahar, Fatma Sarıdağ, Esra Yıldızhan

**Affiliations:** 1 Division of Intensive Care Unit, Department of Internal Medicine, Kayseri City Education and Research Hospital, 38020 Kayseri, Türkiye; marufboran@gmail.com; 2 Department of Internal Medicine, Kayseri City Education and Research Hospital, 38020 Kayseri, Türkiye; kelifbahar@hotmail.com (K.E.B.); fatmasaridag34@gmail.com (F.S.); 3 Division of Hematology, Department of Internal Medicine, Kayseri City Education and Research Hospital, 38020 Kayseri, Türkiye; esra3yildizhan@hotmail.com

**Keywords:** thrombocytopenia, critical care, mortality

## Abstract

**Background/Objectives:** Thrombocytopenia (TP) is common in critically ill patients, but the significance of its severity, timing, persistence, and admission subthreshold platelet count (PC) remains uncertain. We evaluated these associations with mortality in a medical ICU. **Methods:** In this prospective cohort study, 280 adults admitted to a tertiary medical ICU (September 2023–August 2024) were followed until ICU discharge or death. TP was defined as a PC < 150 × 10^9^/L, and admission STP as a first ICU PC of 150–199 × 10^9^/L, irrespective of later platelet course. Outcomes included mortality (ICU, hospital, 28-day, 90-day), organ support, and major complications. **Results:** TP occurred in 118 patients (42.1%), with higher crude mortality at all time points (all *p* < 0.001). In the lagged time-dependent Cox analysis, ICU-acquired TP was associated with ICU mortality in the minimally adjusted model (HR 1.93, CI 1.12–3.35; *p* = 0.018) but not after adjustment for invasive MV (aHR 1.31, CI 0.72–2.40; *p* = 0.372). Admission TP was not significantly associated with ICU mortality in the adjusted model (aHR 1.22, CI 0.74–2.00; *p* = 0.436). Admission PC showed limited discrimination (AUC 0.583, CI 0.508–0.657). No statistically significant difference was found between admission STP and PC ≥ 200 × 10^9^/L (exploratory comparison, wide CIs). **Conclusions**: TP was associated with disease severity and worse crude outcomes, but adjusted longitudinal analyses did not establish an independent association with ICU mortality. No statistically significant difference was found for admission STP versus PC ≥ 200 × 10^9^/L in this exploratory comparison; this should not be interpreted as clinical equivalence or as indicating no prognostic value.

## 1. Introduction

Beyond their role in hemostasis, platelets participate in immune regulation, inflammation, and endothelial interactions [1]. A decrease in platelet count (PC) is defined as thrombocytopenia (TP), and although previous publications have used different threshold values (≤200 × 10^9^/L, ≤80 × 10^9^/L, ≤60 × 10^9^/L) and rates of decrease (<30%, 50%) [2], the current literature defines TP as a PC below 150 × 10^9^/L [3]. It is quite common in intensive care units (ICUs); reported prevalence rates vary considerably across studies (8–56%) [2,3,4]. Many causes of TP, such as sepsis, medications, trauma, and hemodilution, may coexist in critically ill patients [5,6]. Among these etiologies, sepsis remains the most important cause of TP in the ICU and has led to the widespread use of the term “sepsis-associated thrombocytopenia” in the literature [7,8].

TP has been associated with bleeding, length of stay (LOS) in the ICU/hospital, and the need for life support devices, vasopressors, and mortality [5,9,10]. A linear relationship has been found between the severity of TP and mortality and disease severity [11,12]. Moreover, TP lasting longer than three days has been associated with poor prognosis and mortality [8]. Particularly in severe TP, fear of bleeding leads to delay in invasive procedures, postponement of prophylactic anticoagulation, and platelet transfusions [5,13,14]. Correcting one factor in the treatment of TP patients may not always lead to clinical improvement [11]. The most important aspect of treatment is to correct the underlying disease, if possible.

TP may be present at admission to the ICU or may develop during the course of treatment. The development of TP should be considered a consequence of the severity of the disease and the critical condition [15]. Therefore, monitoring the course of PC and the duration of TP is important in clinical follow-up.

Building on previous studies of TP timing and persistence [8,16], our primary objective was to evaluate the associations of admission and ICU-acquired TP with ICU mortality using time-dependent Cox regression. Secondary analyses examined persistent TP and whether admission STP (PC 150–199 × 10^9^/L) was associated with adverse outcomes compared with PC ≥ 200 × 10^9^/L.

## 2. Materials and Methods

### 2.1. Study Design and Population

This prospective observational cohort study was conducted in accordance with the STROBE Statement [17] for cohort studies. The study was conducted at Medical Intensive Care Unit-3 of Kayseri City Education and Research Hospital in Kayseri, Turkey. All consecutive patients aged 18 years and older admitted to the ICU and followed for more than 24 h were eligible for inclusion in the study and had no missing critical information (death, organ failure, need for life support devices, need for blood product replacement) in their records. Patients with known terminal-stage solid or hematological malignancies, patients undergoing active chemotherapy, patients with end-stage (Child–Pugh class C) liver cirrhosis, and patients with uncontrolled bleeding during hospitalization were excluded from the study at the outset because they were associated with platelet abnormalities, regardless of their critical illness status. Inclusion of such patients could confound platelet kinetics and outcome analyses (Figure 1). Of the 415 patients evaluated, 280 met the predefined inclusion criteria and were enrolled. Patients were followed until they were discharged from the hospital or for 90 days. The study was approved by the Institutional Ethics Committee (protocol number 2023–877).

Due to its single-center design and predefined exclusion criteria, selection bias cannot be entirely eliminated, and the findings may not be generalizable to all ICU populations, particularly solid malignancies or hematology-focused cohorts.

### 2.2. Study Procedure

Patient eligibility was assessed upon admission to the ICU. Patients who met the inclusion criteria were informed about the study and enrolled on the same day. In line with the study objectives, standard ICU management protocols were applied without modification.

### 2.3. Sample Size

An a priori sample-size calculation was performed using G*Power version 3.1 for a two-sided Cox proportional hazards regression analysis. Based on previously published ICU TP studies [5] and pre-study institutional mortality data, we assumed an overall ICU mortality rate of 35%, a TP prevalence of approximately 40%, and a hazard ratio of 2.0 for the association between TP and ICU mortality, corresponding to an anticipated ICU mortality of approximately 40% among patients with TP versus 20% among those without. With a two-sided significance level of 0.05, 80% power, and an assumed squared multiple correlation of 0.10 between the exposure and the remaining covariates, approximately 217 patients were required. The required sample size was exceeded, with a total of 280 patients, including 95 ICU deaths, ultimately analyzed, providing adequate statistical power for the primary Cox regression analysis.

### 2.4. Study Data

Clinical data were obtained from patient medical records and the hospital electronic medical record system (KEYDATA). The variables collected included demographic characteristics, comorbidities, reason for admission to the ICU, disease severity scores, therapeutic interventions, transfusion requirements, laboratory parameters, and clinical outcomes. There were no missing data for the outcomes or covariates included in the Cox regression models; therefore, no imputation was required. The APACHE II Score was calculated based on the worst values recorded within the first 24 h of admission to the ICU. The SOFA score was calculated based on the values at the time of admission to the ICU. Recorded therapeutic interventions included anticoagulant and corticosteroid use, vasopressor support (VS), central venous catheter (CVC) placement, mechanical ventilation (MV), renal replacement therapy (RRT), and transfusion data. Laboratory variables included complete blood count (CBC) parameters, biochemical markers, inflammatory markers, coagulation parameters, and arterial blood gas measurements. Platelet counts were measured using impedance-based analyzers and verified by optical methods when necessary. Peripheral blood smears were examined when clinically indicated.

### 2.5. Platelet Measurements

The admission PC was defined as the first complete blood count obtained immediately upon ICU admission. This sample was collected before any platelet transfusion, blood-product replacement, or ICU procedure. None of the included patients had received platelet transfusion in the emergency department before ICU admission. PCs were obtained at least daily, and when more than one measurement was available on the same ICU day, the first measurement was used. Exact intraday timestamps were not included in the analytical dataset. Therefore, a one-day lag was applied to ICU-acquired thrombocytopenia: patients remained unexposed throughout the ICU day on which the first qualifying platelet count was recorded, and exposure began on the following ICU day. Deaths or ICU discharges occurring on the detection day were retained in the analysis and classified as occurring while unexposed. If a substantial discrepancy was identified between automated measurements or the PC was considered clinically inconsistent, the result was verified using the optical PC and peripheral blood smear, and the verified value was used. For patients receiving platelet transfusion, the PC obtained before transfusion was used in platelet classification.

Pseudothrombocytopenia was assessed using a standardized verification procedure. Unexpected or discordant low platelet counts were re-evaluated by optical measurement and examination of a peripheral blood smear for platelet clumping. Values considered attributable to pseudothrombocytopenia were not used for classification. Only the first ICU admission of each patient during the study period was included; ICU readmissions were excluded.

Serial platelet counts obtained from ICU admission until ICU discharge or death were used to assess TP severity, timing, persistence, and recovery. Severity was determined by the nadir platelet count, duration was assessed using consecutive daily measurements, and recovery was defined as the first subsequent PC ≥ 150 × 10^9^/L. Because daily platelet counts were available for all included patients, no interpolation or imputation of daily measurements was required.

### 2.6. Definitions

*Thrombocytopenia (TP)* was defined as a PC < 150 × 10^9^/L [2]. For descriptive longitudinal analyses, *subthreshold thrombocytopenia (STP)* was defined as any PC < 200 × 10^9^/L during ICU follow-up; therefore, this definition could include patients who also met the conventional TP criterion. *Admission STP* was defined as a first PC of 150–199 × 10^9^/L obtained at ICU admission (i.e., without conventional TP at admission). Patients were classified according to the admission value alone; subsequent development of TP (<150 × 10^9^/L) during the ICU stay was not an exclusion criterion. *TP severity* was classified according to the nadir PC during the ICU stay as mild (100–149 × 10^9^/L), moderate (50–99 × 10^9^/L), severe (20–49 × 10^9^/L), or very severe (<20 × 10^9^/L) [16,18]. *Admission TP* was defined as TP present at ICU admission. *ICU-acquired thrombocytopenia (ICU-TP)* was defined as a new PC < 150 × 10^9^/L developing after ICU admission in patients without TP at admission. Patients with TP present at admission or developing during the ICU stay were classified as having TP during the ICU stay, whereas patients who never had a PC < 150 × 10^9^/L were classified as having no TP. *Persistent TP* was defined as a PC < 150 × 10^9^/L on at least three consecutive ICU days (duration ≥ 3 days), whereas transient TP was defined as a duration of less than three days [8]. Patients who died or were discharged before three ICU days could not fulfill the persistence criterion, creating a potential risk of survivor bias in duration-based analyses.

*Bleeding* events were graded according to World Health Organization criteria [19]. *Sepsis* was defined using Sepsis-3 criteria [12]. *Acute kidney injury (AKI)* was defined according to KDIGO guidelines [20]. *Acute liver injury (ALI) and Acute liver failure (ALF)* were defined as previously described [21,22]. *Disseminated Intravascular Coagulation (DIC)* was defined based on International Society of Thrombosis and Haemostasis (ISTH) Scientific and Standardization Committee criteria [23]. Blood and blood product *transfusions* followed current international guidelines [24,25]. *Heparin-induced thrombocytopenia (HIT)* was assessed using the 4T clinical probability score [26].

### 2.7. Outcomes

The primary inferential analysis evaluated the associations of admission TP and ICU-acquired TP with ICU mortality. ICU mortality was defined as death occurring during the index ICU stay before ICU discharge. Hospital mortality was defined as death before final discharge from the index hospitalization. Patients discharged alive from the ICU but remaining hospitalized were followed until final hospital discharge; any recurrent ICU admission during the same hospitalization was included in this follow-up. Secondary analyses evaluated TP severity, persistence, and STP in relation to ICU, hospital, 28-day, and 90-day mortality, bleeding, thrombotic events, transfusion requirements, and organ support during the ICU stay. In-hospital vital status (ICU and hospital mortality) was ascertained from the hospital electronic medical record system. For patients discharged alive from the hospital, vital status at 28 and 90 days after ICU admission was ascertained through the Turkish national death notification system (Ministry of Health), which records the date of death for all citizens nationwide. This system was queried for all enrolled patients, and vital status at both time points was available for the entire cohort, with no loss to follow-up.

### 2.8. Statistical Analysis

Statistical analyses were performed using IBM SPSS Statistics for Windows, Version 27.0 (IBM Corp., Armonk, NY, USA). Normality was assessed using the Shapiro–Wilk test and visual inspection of histograms and Q–Q plots. Continuous variables were summarized as mean ± standard deviation or median (interquartile range), as appropriate. Comparisons between two groups were performed using Student’s *t*-test or the Mann–Whitney U test, whereas comparisons across the three mutually exclusive admission platelet-count categories were performed using the Kruskal–Wallis test. Categorical variables were compared using Pearson’s chi-square test or Fisher’s exact test, as appropriate. ICU mortality proportions across TP severity categories were compared using Pearson’s chi-square test. In addition, pairwise comparisons between admission STP (150–199 × 10^9^/L) and PC ≥ 200 × 10^9^/L were performed as exploratory analyses (Supplementary Table S3). Categorical outcomes were compared using Pearson’s chi-square test (Fisher’s exact test when expected counts were <5) and are reported as risk differences with Newcombe 95% CIs and odds ratios with 95% CIs. Continuous variables were compared using the Mann–Whitney U test. Multiplicity was addressed using the Holm procedure across all 16 pairwise comparisons. Non-significant results were not interpreted as evidence of clinical equivalence, and no equivalence testing was performed.

The primary inferential analysis evaluated the associations of admission TP and ICU-acquired TP with ICU mortality using time-dependent Cox proportional hazards regression. ICU admission was the time origin; ICU death was the event, and discharge alive from the ICU was treated as censoring. admission TP was entered as a fixed baseline covariate. Among patients without TP at admission, ICU-acquired TP was modeled as a time-dependent covariate using this lagged daily exposure definition. Patients who never developed TP remained unexposed. Any TP was not entered as a fixed covariate. The proportional-hazards assumption was assessed by adding covariate-by-log (follow-up time) interaction terms to the time-dependent Cox regression model for the time-varying TP exposure and all model covariates. A statistically significant interaction was considered evidence of non-proportionality, and the affected covariate was handled by stratification in the final model.

Model 1 was a minimally adjusted (exposure-only) model that included admission TP as a fixed baseline covariate and ICU-TP as a time-dependent covariate with a one-day lag; each exposure was therefore mutually adjusted for the other, but not for clinical covariates. Model 2 additionally included centered linear and quadratic APACHE II terms, sepsis, and vasopressor use. APACHE II was calculated using the worst values within the first 24 h; sepsis and vasopressor use were assessed at ICU admission. Invasive mechanical ventilation at ICU admission was included as a stratification variable based on evidence of non-proportional hazards in the initial assessment. Covariates were selected on clinical grounds. The functional form of APACHE II was evaluated in the initial analyses using likelihood-ratio tests comparing linear and linear-plus-quadratic specifications. APACHE II was centered at 25 before calculating the quadratic term. The centered linear and quadratic APACHE II terms selected in the initial analyses were retained in the revised models.

Duration-based analyses were restricted to patients for whom the TP duration could be determined. Twelve patients who died or were discharged before three ICU days could not be classified as persistent or transient. These analyses were considered exploratory because of potential survivor bias. In a separate time-dependent analysis, patients remained unexposed until they fulfilled the prespecified persistence criterion and were considered exposed thereafter. Patients who died or were discharged before fulfilling this criterion were retained in the analysis and contributed unexposed follow-up time until ICU death or discharge.

Unadjusted comparisons presented in Tables 2 and 3 and Supplementary Table S2 were exploratory. No formal multiplicity adjustment was applied; therefore, *p*-values from these analyses are nominal and should be interpreted cautiously. Primary inferences were based on adjusted Cox regression estimates reported with 95% confidence intervals. Influential observations were evaluated using DFBETA diagnostics, and sensitivity analyses were performed by excluding observations with the largest absolute DFBETA values one at a time and refitting the same models.

ROC analysis evaluated the discriminatory ability of admission PC for ICU mortality. The AUC was reported with its 95% confidence interval. Sensitivity and specificity were assessed at admission platelet thresholds of <150 and <200 × 10^9^/L, with 95% confidence intervals calculated using the Wilson score method. Wilson 95% confidence intervals were also calculated for mortality proportions in Supplementary Table S1. The Cox models were designed to estimate adjusted associations rather than to develop a clinical prediction model; therefore, model discrimination, calibration, and internal validation were not assessed. A two-sided *p*-value < 0.05 was considered statistically significant.

## 3. Results

### 3.1. Study Population

A total of 280 patients were included in the study. The median age was 72 years (IQR 61–82). At ICU admission, the median Glasgow Coma Scale (GCS), APACHE II, and SOFA scores were 13 (IQR 10–15), 21 (IQR 15–29), and 5 (IQR 3–8), respectively. The most common comorbidities were hypertension, diabetes mellitus, and chronic kidney disease. ICU mortality was 33.9%, whereas hospital, 28-day, and 90-day mortality rates were 38.9%, 31.8%, and 42.5%, respectively. Vital status at 28 and 90 days was available for all 280 patients (0% loss to follow-up). Reasons for ICU admission were available for all 280 patients. Because multiple admission diagnoses could be recorded for a single patient, the percentages were not mutually exclusive. The most common reasons for ICU admission were sepsis (58.2%), gastrointestinal bleeding (12.5%), acute kidney injury (9.3%), acute decompensated heart failure or volume overload (8.9%), major electrolyte disturbances (6.8%), postoperative care (6.1%), diabetic ketoacidosis (5.7%), and post-cardiac-arrest care (5.0%). No ICU or hospital deaths were attributed to hemorrhage. Baseline characteristics of the study population are summarized in Table 1.

### 3.2. Comparison According to the Presence of Thrombocytopenia

Patients with TP had lower GCS scores and higher APACHE II and SOFA scores than those without TP (all *p* < 0.001), indicating greater illness severity. They also had significantly higher ICU, hospital, 28-day, and 90-day mortality rates (all *p* < 0.001) and a longer ICU length of stay (*p* < 0.001). In addition, patients with TP had significantly lower lymphocyte counts, hemoglobin levels, and fibrinogen concentrations, whereas AST, LDH, total bilirubin, creatinine, INR, D-dimer, lactate, and procalcitonin levels were significantly higher. Comparisons between patients with and without TP are presented in Table 2.

### 3.3. Discrimination of ICU Mortality by Admission PC

Admission PC showed limited discrimination for ICU mortality (AUC 0.583, 95% CI 0.508–0.657; *p* = 0.023; Figure 2). At admission, PC thresholds were <150 and <200 × 10^9^/L, sensitivity for ICU mortality was 34.7% (95% CI 25.9–44.7%) and 47.4% (95% CI 37.6–57.3%), respectively, while specificity was 84.3% (95% CI 78.4–88.9%) and 59.5% (95% CI 52.3–66.3%), respectively.

### 3.4. Admission Platelet-Count Categories and Subthreshold TP

Based on the first PC obtained at ICU admission, 160 patients (57.1%) had a PC ≥ 200 × 10^9^/L, 58 (20.7%) had admission STP (150–199 × 10^9^/L), and 62 (22.1%) had conventional TP (<150 × 10^9^/L). ICU-acquired TP subsequently developed in 25 of 58 patients (43.1%) with admission STP and in 31 of 160 patients (19.4%) with an admission PC ≥ 200 × 10^9^/L. Overall comparisons across these mutually exclusive groups showed significant differences in APACHE II and SOFA scores, ICU, hospital, 28-day, and 90-day mortality, transfusion requirements, MV, VP requirement, and DIC, whereas the overall difference in AKI was borderline (*p* = 0.050). The significant overall differences were primarily driven by the poorer clinical characteristics and outcomes of patients with conventional TP (Table 3).

In pairwise comparisons, admission STP did not differ significantly from PC ≥ 200 × 10^9^/L in any outcome (Supplementary Table S3). Point estimates for mortality and organ support were numerically lower in the admission STP group (e.g., ICU mortality 20.7% vs. 31.3%; risk difference −10.6 percentage points, 95% CI −21.9 to 3.2), and no comparison remained significant after Holm adjustment. However, confidence intervals were wide.

### 3.5. Impact of TP Duration on Mortality

Crude ICU mortality was higher among patients who developed persistent TP than among those who did not meet the persistence criterion (42/79 [53.2%] vs. 53/201 [26.4%]; *p* < 0.001). However, this comparison did not account for the time required to complete three consecutive days of TP. In the time-dependent Cox analysis, persistent TP was not significantly associated with ICU mortality in Model 1 (HR 1.14, 95% CI 0.73–1.78; *p* = 0.574). The association remained nonsignificant after adjustment for admission TP, APACHE II modeled with linear and quadratic terms, and sepsis, invasive MV, and VP use at ICU admission (aHR 0.66, 95% CI 0.36–1.20; *p* = 0.172). VP use at ICU admission was associated with a higher hazard of ICU mortality in the adjusted model (aHR 2.48, 95% CI 1.45–4.24; *p* < 0.001). Both models included 280 patients and 95 ICU deaths (Table 4).

In two separate sensitivity analyses excluding influential observations one at a time, the aHR for persistent TP was 0.60 (95% CI 0.33–1.10; *p* = 0.098) and 0.50 (95% CI 0.27–0.94; *p* = 0.030), respectively. Admission TP became statistically significant in the first sensitivity analysis (aHR 1.99, 95% CI 1.09–3.64; *p* = 0.025). APACHE II term also varied across these analyses (likelihood-ratio *p* = 0.006 and *p* = 0.117, respectively). In the adjusted persistent TP model, the coefficient for the centered linear APACHE II term was 0.017 (SE 0.014; *p* = 0.228), and that for the quadratic term was 0.002 (SE 0.001; *p* = 0.021).

### 3.6. Association Between TP Severity and Mortality

Mortality rates increased progressively with increasing TP severity. ICU mortality rose from 27.3% in patients with mild TP to 40.9% in those with moderate TP and reached 84.2% and 90.9% in patients with severe and very severe TP, respectively (*p* < 0.001). A similar pattern was observed for hospital, 28-day, and 90-day mortality. The relationship between TP severity and mortality outcomes is presented in Supplementary Table S1.

### 3.7. Comparison Between Admission and ICU-Acquired TP

Patients with admission TP and those who developed ICU-acquired TP (ICU-TP) had comparable demographic and baseline clinical characteristics. Duration-based TP (≥3 days) could be evaluated in 106 of the 118 patients with TP; 12 patients were excluded because TP duration could not be adequately assessed due to an ICU stay of <3 days or early death. Among patients whose TP duration could be classified, persistent TP occurred in 49 of 56 patients with admission TP (87.5%) and 30 of 50 patients with ICU-acquired TP (60.0%; *p* = 0.003). Patients with ICU-TP had higher WBC and neutrophil counts and higher lactate levels, whereas lymphocyte counts and platelet counts were lower in the admission TP group. Demographic, clinical, and laboratory characteristics are presented in Table 5.

### 3.8. Transfusion Requirements, Complications and Clinical Interventions

Patients who developed TP at any time during the ICU stay more frequently required platelet, RBC, and FFP/cryoprecipitate transfusions than patients who never developed TP (all *p* < 0.001). Sepsis, DIC, AKI, and ALI were also more common among patients who developed TP at any time (all *p* < 0.001), whereas the rates of hemorrhage and thrombosis were similar between groups. These patients also required more intensive organ support, including RRT, invasive MV, and VP therapy (all *p* < 0.001). Steroid therapy and CVC placement were more frequent among patients with TP, whereas thromboprophylaxis and antibiotic use did not differ significantly. Detailed comparisons of transfusion requirements, complications, and clinical interventions are presented in Supplementary Table S2.

### 3.9. Time-Dependent Cox Regression Analysis

In the minimally adjusted model (Model 1), lagged ICU-acquired TP was associated with a higher hazard of ICU mortality (HR 1.93, 95% CI 1.12–3.35; *p* = 0.018), whereas admission TP was not significantly associated with ICU mortality (HR 1.44, 95% CI 0.89–2.34; *p* = 0.140). In the final lagged time-dependent Cox model, ICU-acquired TP was not significantly associated with ICU mortality (aHR 1.31, 95% CI 0.72–2.40; *p* = 0.372). Admission TP was also not significantly associated with ICU mortality (aHR 1.22, 95% CI 0.74–2.00; *p* = 0.436) (Table 6). These adjusted estimates apply only to patients with ICU follow-up exceeding 24 h, reflect partial overlap between the first-24 h APACHE II assessment window and early TP onset, and incorporate a one-day lag applied to ICU-acquired TP because intraday platelet timestamps were unavailable (see Limitations).

In the initial proportional-hazards assessment, invasive mechanical ventilation at ICU admission showed evidence of non-proportional hazards. This variable was therefore used for stratification in the revised Cox model incorporating a one-day lag for ICU-acquired TP.

In a sensitivity analysis excluding one influential observation identified by DFBETA diagnostics, the adjusted estimates moved toward the null and remained statistically non-significant for both ICU-acquired TP (aHR 1.10, 95% CI 0.60–2.04; *p* = 0.758) and admission TP (aHR 1.04, 95% CI 0.62–1.73; *p* = 0.881). This analysis included 279 patients and 94 ICU deaths. The full-sample model, including 280 patients and 95 ICU deaths, was retained as the primary analysis.

## 4. Discussion

### 4.1. Main Findings

In this single-center prospective cohort study, we evaluated the clinical significance of TP in critically ill patients according to its presence, severity, timing, and duration during the ICU stay. First, the association between ICU-acquired TP and ICU mortality was attenuated and no longer statistically significant after multivariable adjustment, while admission TP was not significantly associated with ICU mortality in either model. Second, patients with TP had higher crude ICU, hospital, 28-day, and 90-day mortality rates than those without TP. Third, mortality generally increased with increasing TP severity, with the highest rates observed in the severe and very severe categories. Fourth, persistent TP was associated with worse crude clinical outcomes in descriptive analyses, whereas persistence was not independently associated with ICU mortality in the time-dependent analysis. Finally, admission STP (150–199 × 10^9^/L) was not significantly associated with greater disease severity or adverse outcomes compared with admission platelet counts ≥ 200 × 10^9^/L. Together, these findings provide exploratory information on the intermediate admission platelet-count range and the timing and persistence of TP.

### 4.2. TP as a Marker of Disease Severity

Patients with TP had greater disease severity, higher crude mortality rates, and more frequent organ-support requirements. In the time-dependent minimally adjusted model 1 that included both admission and ICU-acquired TP, ICU-acquired TP was associated with an approximately twofold higher hazard of ICU mortality. This association was attenuated and was no longer statistically significant after adjustment for APACHE II assessed within the first 24 h and sepsis, invasive MV, and VP use at ICU admission. These findings suggest that the observed association may partly reflect underlying illness severity and organ-support requirements. However, the confidence intervals do not exclude clinically relevant associations, and the adjusted results should not be interpreted as evidence of no association. These findings are consistent with those reported in large international cohort studies [16].

### 4.3. Pathophysiological Mechanisms and Inflammatory Response

Interestingly, conventional inflammatory markers such as NLR and CRP did not differ significantly between groups, suggesting that routine inflammatory indices may not fully capture the complex inflammatory processes underlying TP in critically ill patients. In contrast, the laboratory profile observed in patients with TP was consistent with more severe coagulopathy and tissue hypoperfusion, as reflected by higher INR, D-dimer, and lactate levels together with lower fibrinogen concentrations. These findings support the concept that TP in critical illness primarily reflects platelet consumption secondary to systemic inflammation and coagulation activation, particularly in the setting of sepsis and disseminated intravascular coagulation. Beyond their role in hemostasis, platelets are increasingly recognized as key mediators of immune regulation, inflammation, and endothelial function. Accordingly, TP likely represents the complex interplay between inflammation, coagulation, and endothelial dysfunction rather than an isolated hematological abnormality [2].

### 4.4. Timing and Duration of TP

Persistent TP has previously been associated with poor outcomes in critically ill patients [8]. However, classification as persistent requires patients to remain alive and under observation long enough to fulfill the specified duration criterion. In the present study, persistent TP was therefore evaluated as a time-dependent exposure. After accounting for the time required to fulfill the three-day criterion, persistent TP was not associated with ICU mortality in either the unadjusted or adjusted analysis. However, this estimate was unstable across leave-one-out sensitivity analyses (aHR range 0.50–1.99), so these findings should be considered exploratory and hypothesis-generating. More broadly, direct comparisons between persistent and transient TP may be affected by survival bias and should be interpreted cautiously.

The attenuation of the ICU-acquired TP association after adjustment should be interpreted in the context of baseline illness severity and admission clinical factors. The confidence intervals do not exclude clinically relevant associations.

### 4.5. Platelet Thresholds and Subthreshold TP

The definition of TP varies considerably across the literature, and no universally accepted diagnostic threshold exists. Although several absolute PC and relative platelet decline criteria have been proposed, TP is most commonly defined as a PC < 150 × 10^9^/L in contemporary clinical practice and published studies [11].

To examine whether PCs above the conventional TP threshold were clinically informative, we evaluated admission STP (150–199 × 10^9^/L) using mutually exclusive categories based on the first PC obtained at ICU admission. Although the threshold of 200 × 10^9^/L was based on a previously proposed definition [2], patients with admission STP showed no statistically significant differences from those with platelet counts ≥ 200 × 10^9^/L in disease severity, mortality, organ-support requirements, transfusion requirements, or major complications in pairwise comparisons (Supplementary Table S3). Because these comparisons were exploratory, the confidence intervals were wide, and the study was not designed to test equivalence, these findings should not be interpreted as evidence that the two groups are clinically equivalent. The significant overall differences across the three admission platelet-count categories were predominantly attributable to the conventional TP group.

These findings highlight the importance of distinguishing admission STP from conventional TP when evaluating platelet thresholds. Patients with admission STP were more likely to develop TP later during the ICU stay than those with PC ≥ 200 × 10^9^/L (43.1% vs. 19.4%), likely reflecting their proximity to the conventional threshold. Nevertheless, no statistically significant difference in crude outcomes was found compared with patients with PC ≥ 200 × 10^9^/L. Given the exploratory nature of this comparison and the wide confidence intervals, this finding should not be interpreted as evidence that this admission range is clinically equivalent to PC ≥ 200 × 10^9^/L or that it lacks prognostic value. The proposed STP threshold should therefore be interpreted cautiously and requires further evaluation in larger prospective studies.

### 4.6. Strengths and Limitations of the Study

Several limitations should be acknowledged. First, this was a single-center study, and the exclusion of patients with advanced cirrhosis, hematological malignancies, active chemotherapy, and uncontrolled major bleeding may limit the generalizability of the findings. The results primarily reflect a medical ICU population and may not apply to mixed surgical ICUs or specialized populations in which the mechanisms of TP may differ. Second, as an observational study, residual confounding and selection bias cannot be completely excluded despite the prospective design and systematic data collection. Specifically, requiring ICU follow-up exceeding 24 h conditioned inclusion on survival and continued ICU stay during this initial period, introducing potential selection and guarantee-time bias. This criterion excluded 54 patients: 26 who died, 27 who were transferred to a ward, and one who was transferred to another ICU within the first 24 h. Excluding early deaths and early ward transfers removes patients with different clinical trajectories; therefore, the net direction and magnitude of bias in the estimated associations between TP and mortality remain uncertain. The findings should consequently be interpreted as applying to patients with ICU follow-up exceeding 24 h. Admission STP was defined by the admission PC alone, and 43.1% of these patients subsequently developed conventional TP, which may have diluted differences relative to patients with PC ≥ 200 × 10^9^/L. More broadly, the study was not powered for pairwise comparisons between platelet-count categories or for equivalence testing. Finally, analyses involving ICU-acquired and persistent TP may be affected by time-dependent and survival biases. ICU-acquired TP was modeled using a one-day lag because exact intraday timestamps were unavailable in the analytical dataset. This avoided assigning pre-measurement time to the exposed state, but also classified any post-measurement time on the detection day as unexposed. Consequently, within-day temporal uncertainty could not be fully resolved. The first 24 h APACHE II assessment window may overlap with early TP onset, limiting its interpretation as a strictly baseline adjustment variable. Persistent TP required three consecutive ICU days with a PC < 150 × 10^9^/L. Patients who died or were discharged before fulfilling this criterion could not be classified in the descriptive duration analysis, but they were retained in the time-dependent analysis and contributed unexposed follow-up time. Therefore, findings related to TP timing and duration should be considered exploratory and interpreted cautiously. In the persistent TP model, effect estimates and the evidence supporting the quadratic APACHE II term were sensitive to individual observations. These findings warrant cautious interpretation and do not establish a protective effect of persistent TP. The multivariable models were designed to estimate adjusted associations rather than to develop a clinical prediction model; therefore, discrimination, calibration, and internal validation were not assessed, and the adjusted estimates should not be interpreted as validated individual mortality predictions. Although daily platelet counts were available, relative platelet decline, rates of change, and longitudinal trajectories were not analyzed; thus, clinically relevant changes above the conventional TP threshold may not have been captured.

## 5. Conclusions

TP was common among critically ill patients and was associated with greater illness severity, more frequent organ-support requirements, and higher crude mortality. ICU-acquired TP was associated with an increased hazard of ICU mortality in the minimally adjusted (exposure-only) time-dependent model, but this association was attenuated after adjustment for baseline illness severity and admission clinical factors. Greater TP severity and persistent TP (≥3 days) were associated with worse crude clinical outcomes; however, these findings should be interpreted cautiously because of potential survivor bias, and persistent TP did not show a statistically significant independent association with ICU mortality in the time-dependent analysis. Overall, TP dynamics were associated with markers of clinical deterioration in descriptive analyses, whereas the adjusted analyses did not establish an independent association with ICU mortality. No statistically significant difference in clinical outcomes was found between admission STP (a first ICU PC of 150–199 × 10^9^/L) and an admission PC ≥ 200 × 10^9^/L in this exploratory comparison. Given the wide confidence intervals, this finding should not be interpreted as evidence that the two groups are clinically equivalent or that the 200 × 10^9^/L threshold lacks prognostic value; larger studies are needed to evaluate this threshold more precisely.

## Figures and Tables

**Figure 1:**
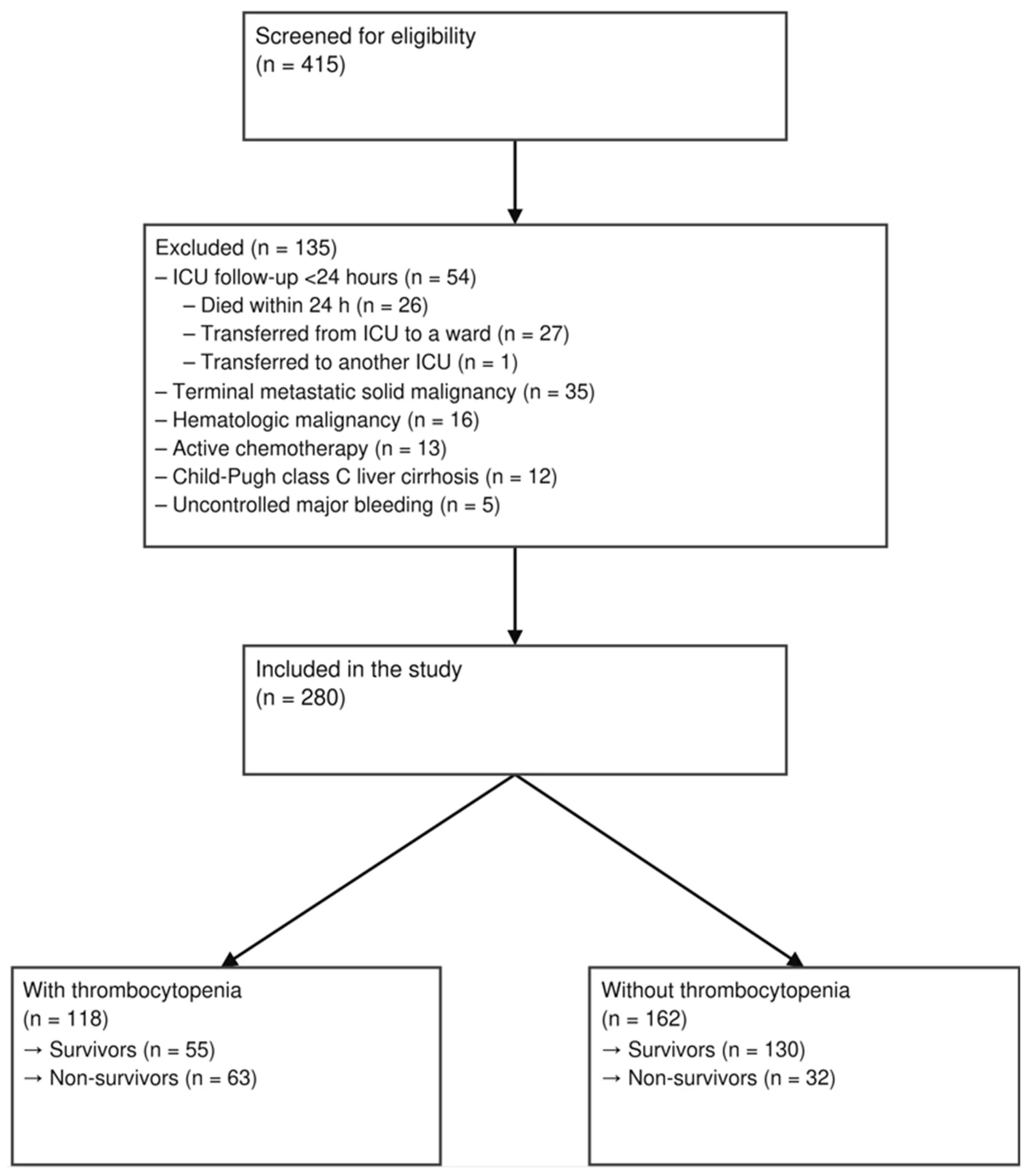
Flow diagram of patient selection and study group classification.

**Figure 2:**
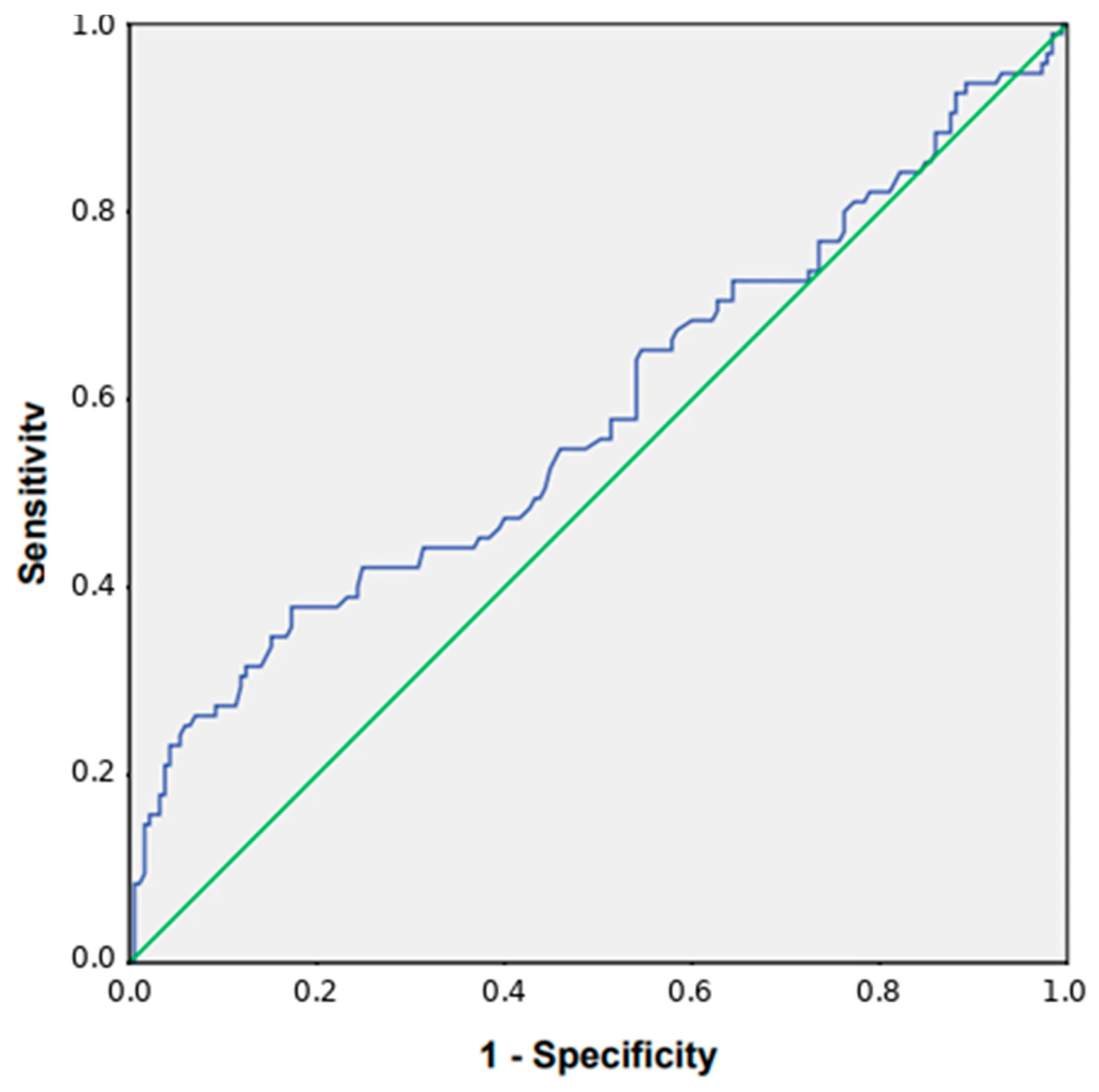
ROC Curve of Admission Platelet Count for ICU Mortality.

**Table 1:** Overall Study Population Characteristics and Clinical Outcomes.

Variable	Value
Age, (years)	72 (61–82)
GCS score	13 (10–15)
APACHE II score	21 (15–29)
SOFA score	5 (3–8)
Sepsis yes n (%)	165 (58.9)
Comorbidities n (%)	
Hypertension	139 (49.6)
Diabetes mellitus	115 (41.1)
Chronic kidney disease	63 (22.5)
ICU admission n (%)	
Emergency Department	186 (66.4)
Ward	70 (25)
Other ICU	24 (8.6)
ICU mortality n (%)	95 (33.9)
Hospital mortality n (%)	109 (38.9)
28-day mortality n (%)	89 (31.8)
90-day mortality n (%)	119 (42.5)
ICU days	5 (3–10)
Hospital days	11 (7–26)
Admission TP n (%)	62 (22.1)
ICU-TP n (%)	56 (20)
No TP during ICU stay n (%)	162 (57.9)
TP duration ≥ 3 days n (%)	79 (28.2)
STP * at admission n (%)	120 (42.9)
STP * developed in the ICU n (%)	76 (27.1)
STP * never n (%)	84 (30)

Abbreviations: APACHE II, Acute Physiology and Chronic Health Evaluation II; GCS, Glasgow Coma Scale; ICU, Intensive Care Unit; ICU-TP, ICU-acquired thrombocytopenia; SOFA, Sequential Organ Failure Assessment; STP, Subthreshold Thrombocytopenia; TP, Thrombocytopenia. * This STP definition describes PC below 200 × 10^9^/L, including conventional TP (<150 × 10^9^/L), and should not be interpreted as admission STP. Admission STP (150–199 × 10^9^/L at ICU admission) is evaluated separately in Table 3. Comorbidities are presented as n (%), with percentages calculated using the total cohort (N = 280) as the denominator. Patients could have more than one comorbidity. There were no missing comorbidity data.

**Table 2:** Admission Characteristics, Laboratory Measurements, and Clinical Outcomes According to TP Status During the ICU Stay.

Variables	TP During ICU Stay n = 118	No TP During ICU Stay n = 162	*p*
**Admission characteristics**			
Age	75(64–82)	70 (58–81)	0.072
Female sex n (%)	52 (44.1)	70 (43.2)	0.90
GCS score	12 (7–14)	14 (12–15)	<0.001
APACHE II score	26 (19–33)	19 (13–26)	<0.001
SOFA score	7 (5–10)	4 (2–6)	<0.001
**Laboratory variables**			
WBC count (×10^9^/L)	11.2 (6.5–15.6)	12.4 (9–17.8)	0.026
Lymphocyte count (×10^9^/L)	0.8 (0.53–1.4)	1.2 (0.78–1.8)	<0.001
Neutrophil count (×10^9^/L)	8.9 (5.2–13.6)	10 (6.9–15.3)	0.062
Hemoglobin (g/dL) mean ± SD	11.1 ± 6.7	12 ± 8.2	0.001
Platelet count (×10^9^/L)	143 (100–203)	254 (206–309)	<0.001
NLR	9.6 (4.8–23.1)	9 (4.3–16.6)	0.263
AST (U/L)	31 (17–107)	23 (15–39)	0.005
ALT (U/L)	22 (10–64)	16 (10–31)	0.126
AST/ALT ratio	1.5 (1.1–2.2)	1.3 (0.97–1.8)	0.045
LDH (U/L)	326 (246–468)	278 (221–360)	0.001
Total bilirubin (mg/dL)	0.8 (0.4–1.5)	0.6 (0.3–1)	0.001
Creatinine (mg/dL)	1.65 (0.98–3.3)	1.1 (0.78–2)	0.006
INR	1.35 (1.19–1.8)	1.15 (1.1–1.33)	<0.001
Fibrinogen (mg/L)	3245 (2298–4491)	4400 (3300–5654)	<0.001
D Dimer (μg/L)	5620 (1905–10,412)	2030 (920–5025)	<0.001
pH	7.34 (7.27–7.43)	7.37 (7.27–7.42)	0.689
Lactate (mmol/L)	2 (1.2–3.5)	1.5 (1–2.3)	0.001
PCT (ng/mL)	1 (0.2–5.1)	0.28 (0.1–1.2)	<0.001
CRP (mg/L)	75 (24–155)	59 (11–151)	0.21
**Clinical Outcomes**			
ICU mortality n (%)	63 (53.4)	32 (19.8)	<0.001
Hospital mortality n (%)	70 (59.3)	39 (24.1)	<0.001
28-day mortality n (%)	57 (48.3)	32 (19.8)	<0.001
90-day mortality n (%)	75 (63.6)	44 (27.2)	<0.001
ICU days	6 (3–13)	4 (3–8)	<0.001
Hospital days	13 (8–28)	11 (7–24)	0.10

Abbreviations: APACHE II, Acute Physiology and Chronic Health Evaluation II; GCS, Glasgow Coma Scale; ICU, Intensive Care Unit; TP, Thrombocytopenia; WBC, White Blood Cell. Laboratory measurements were obtained at ICU admission. Comparisons with clinical outcomes are descriptive and do not establish a causal effect of TP. *p* values are unadjusted and relate to exploratory comparisons; they should be interpreted with caution.

**Table 3:** Comparison of Admission Characteristics, Interventions, Complications, and Clinical Outcomes Across Mutually Exclusive Admission Platelet-Count Categories.

Variables	Platelet Count ≥ 200 × 10^9^/L n = 160	Admission STP, 150–199 × 10^9^/L n = 58	TP < 150 × 10^9^/L n = 62	*p*
**Admission characteristics**				
Age	71.5 (57.2–81.7)	74.5 (63–85.2)	73 (64.5–82)	0.357
Female sex n (%)	68 (42.5)	28 (48.3)	26 (41.9)	0.717
GCS score	13 (10–15)	14 (12–15)	12 (8–14)	0.111
APACHE II score	20.5 (15–29)	19.5 (15–26)	25.5 (18–32)	0.014
SOFA score	5 (3–7)	4 (2–6.5)	7 (6–10)	<0.001
**Interventions during the ICU stay**				
Platelet transfusion, yes n (%)	9 (5.6)	3 (5.2)	20 (32.3)	<0.001
RBC Transfusion n (%)	55 (34.4)	17 (29.3)	31 (50)	0.040
FFP/Cryoprecipitate transfusion n (%)	28 (17.5)	9 (15.5)	23 (37.1)	0.003
MV requirement, yes n (%)	54 (33.8)	13 (22.4)	34 (54.8)	<0.001
VP requirement, yes n (%)	55 (34.4)	14 (24)	39 (62.9)	<0.001
**Complications during the ICU stay**				
DIC yes n (%)	16 (10)	9 (15.5)	24 (38.7)	<0.001
AKI yes n (%)	70 (43.8)	20 (34.5)	35 (56.5)	0.05
**Clinical outcomes**				
ICU mortality n (%)	50 (31.3)	12 (20.7)	33 (53.2)	<0.001
Hospital mortality n (%)	58 (36.3)	13 (22.4)	38 (61.3)	<0.001
28-day mortality n (%)	50 (31.3)	11 (19)	28 (45.2)	0.009
90-day mortality n (%)	63 (39.4)	16 (27.6)	40 (64.5)	<0.001

Abbreviations: AKI, Acute Kidney Injury; APACHE II, Acute Physiology and Chronic Health Evaluation II; DIC, Disseminated Intravascular Coagulation; FFP, Fresh Frozen Plasma; GCS, Glasgow Coma Scale; ICU, Intensive Care Unit; RBC, Red Blood Cell; SOFA, Sequential Organ Failure Assessment; STP, Subthreshold Thrombocytopenia. Admission PC categories were defined using the first platelet measurement obtained at ICU admission. Interventions and complications were recorded during the subsequent ICU stay. These comparisons are descriptive and do not establish causality. *p* values are unadjusted and relate to exploratory comparisons; they should be interpreted with caution.

**Table 4:** Time-Dependent Cox Regression Analysis of Persistent TP in Relation to ICU Mortality.

Variable	Model 1 HR (95% CI)	*p*	Model 2 aHR (95% CI)	*p*
Persistent TP *	1.14 (0.73–1.78)	0.574	0.66 (0.36–1.20)	0.172
Admission TP			1.56 (0.88–2.78)	0.130
APACHE II, centered linear term ^			1.017 (0.990–1.045)	0.228
APACHE II, centered quadratic term ^			1.002 (1.000–1.004)	0.021
Sepsis			1.07 (0.53–2.16)	0.846
VP requirement			2.48 (1.45–4.24)	<0.001
MV requirement			0.94 (0.53–1.65)	0.827

Abbreviations: HR, hazard ratio; aHR, adjusted hazard ratio; CI, confidence interval; ICU, intensive care unit; APACHE II, Acute Physiology and Chronic Health Evaluation II; MV, Mechanical Ventilation; TP, Thrombocytopenia; VP, Vasopressor. Model 1 evaluated persistent TP as a time-dependent covariate. Model 2 additionally adjusted for admission TP, APACHE II using linear and quadratic terms, and sepsis, VP use, and invasive MV at ICU admission. APACHE II was calculated using the worst values within the first 24 h. * Patients were considered unexposed until they completed three consecutive ICU days with PC < 150 × 10^9^/L and exposed thereafter. Patients who died or were discharged before fulfilling the three-day criterion remained in the risk set as unexposed until death or ICU discharge. ^ APACHE II was centered at 25 before calculating its square. Because both terms were included, a single constant hazard ratio per APACHE II point is not reported. The linear term had B = 0.017, SE = 0.014, *p* = 0.228; the quadratic term had B = 0.002, SE = 0.001, *p* = 0.021. Adding the quadratic term improved model fit (likelihood-ratio χ^2^ = 4.954, df = 1, *p* = 0.026). The adjusted model included all 280 patients, with 95 ICU deaths and 185 patients censored at ICU discharge. No patients were excluded because of missing data.

**Table 5:** Comparison of Demographic, Clinical, and Laboratory Characteristics Between Patients with Admission TP and ICU-TP.

Variables	Admission TP n = 62	ICU-TP n = 56	*p*
Age	73 (64–82)	76 (64–85)	0.21
Female sex n (%)	26 (41.9)	26 (46.4)	0.74
GCS score	12 (8–14)	12 (4–14)	0.29
APACHE II score	25 (18–32)	26 (20–35)	0.33
SOFA score	7 (6–10)	6 (4–10)	0.17
TP duration ≥ 3 days n (%)	49/56 (87.5)	30/50 (60)	0.003
ICU mortality n (%)	33 (53)	30 (53)	0.97
Hospital mortality n (%)	38 (61)	32 (57)	0.64
28-day mortality n (%)	28 (45)	29 (51)	0.47
90-day mortality n (%)	40 (64)	35 (62)	0.82
ICU days	6 (3–13)	6.5 (3–13.7)	0.96
Hospital days	17 (8–40)	10.5 (7–24)	0.049
**Laboratory variables**			
WBC count (×10^9^/L)	8.7 (5.2–12.6)	14 (10–19.7)	<0.001
Lymphocyte count (×10^9^/L)	0.71 (0.52–1.24)	0.99 (0.53–1.67)	0.02
Neutrophil count (×10^9^/L)	6.7 (4.1–9.8)	11.8 (8.3–16.7)	<0.001
Hemoglobin (g/dL) mean ± SD	10.5 ± 2.2	10.3 ± 3.2	0.80
Platelet count (×10^9^/L)	103 (46–125)	205 (172–237)	<0.001
NLR	9.3 (4.5–17.6)	10.6 (5.2–25)	0.328
AST (U/L)	31 (19–106)	28 (16–107)	0.80
ALT (U/L)	20 (10–56)	22 (10–69)	0.68
AST/ALT ratio	1.5 (1.1–2.3)	1.5 (1.1–2)	0.65
Total bilirubin (mg/dL)	0.85 (0.5–1.6)	0.65 (0.4–1.47)	0.28
Creatinine (mg/dL)	1.79 (0.98–3.3)	1.57 (0.94–3.5)	0.72
INR	1.29 (1.19–1.80)	1.38 (1.2–1.97)	0.54
Fibrinogen (mg/L)	2931 (2004–4344)	3480 (2656–4902)	0.09
D Dimer (μg/L)	6555 (2372–10,107)	5220 (1875–11,192)	0.45
pH	7.38 (7.28–7.44)	7.32 (7.25–7.38)	0.054
Lactate (mmol/L)	1.7 (1.1–2.7)	2.6 (1.3–3.9)	0.02
PCT (ng/mL)	1.27 (0.24–4.55)	1 (0.19–6.6)	0.64
CRP (mg/L)	61 (25–135)	84 (15–175)	0.55

Abbreviations: APACHE II, Acute Physiology and Chronic Health Evaluation II; GCS, Glasgow Coma Scale; ICU, Intensive Care Unit; ICU-TP, ICU-acquired thrombocytopenia; SOFA, Sequential Organ Failure Assessment; TP, Thrombocytopenia; WBC, White blood cell count. Duration classification was available for 56 of 62 patients with admission TP and 50 of 56 patients with ICU-TP. Twelve patients could not be classified as persistent or transient because death or ICU discharge occurred before the duration criterion could be assessed.

**Table 6:** Time-Dependent Cox Regression Analysis of Admission and ICU-Acquired TP in Relation to ICU Mortality.

Variable	Model 1 (Minimally Adjusted) HR (95% CI)	*p*	Model 2 aHR (95% CI)	*p*
Admission TP	1.44 (0.887–2.342)	0.140	1.218 (0.741–2.001)	0.436
Lagged ICU-TP *	1.93 (1.120–3.346)	0.018	1.314 (0.721–2.395)	0.372
APACHE II centered linear term ^			1.009 (0.981–1.038)	0.539
APACHE II, centered quadratic term ^			1.001 (1.000–1.003)	0.114
Sepsis			1.097 (0.548–2.195)	0.793
VP requirement			2.070 (1.193–3.594)	0.010

Abbreviations: APACHE II, Acute Physiology and Chronic Health Evaluation II; CI, confidence interval; HR, hazard ratio; aHR, adjusted hazard ratio; ICU-TP, ICU-acquired thrombocytopenia; MV, Mechanical Ventilation; TP, Thrombocytopenia; VP, Vasopressor. Model 1 (minimally adjusted, exposure-only) included admission TP as a fixed covariate and lagged ICU-acquired TP as a time-dependent covariate, each adjusted for the other. Model 2 additionally included centered linear and quadratic APACHE II terms, sepsis, and vasopressor use at ICU admission. Invasive mechanical ventilation at ICU admission was modeled as a stratification variable because its proportional-hazards assumption was violated. APACHE II was calculated using the worst values during the first 24 h; the other adjustment variables were assessed at ICU admission. ICU admission was the time origin, ICU death was the event, and discharge alive from the ICU was treated as censoring. * Lagged ICU-acquired TP was modeled as a time-dependent covariate. Patients remained unexposed throughout the ICU day on which the first qualifying PC was recorded; exposure began on the following ICU day. If death or ICU discharge occurred on that same day, the day was classified as unexposed. ^ APACHE II was centered at 25 before calculation of the quadratic term. The linear and quadratic terms should be interpreted jointly and do not represent a constant hazard ratio per one-point increase in APACHE II. The primary adjusted model included 280 patients, 95 ICU deaths, and 185 patients censored at ICU discharge. No patients were excluded because of missing data.

## Data Availability

The datasets used and/or analyzed during the current study are available from the corresponding author on reasonable request.

## Supplementary Materials

The following supporting information can be downloaded at https://www.mdpi.com/article/10.3390/jcm15207869/s1, Table S1. Association Between Thrombocytopenia Severity Based on the Nadir ICU Platelet Count and Clinical Outcomes. Table S2. Comparison of Interventions, Complications, and Outcomes Between Patients With and Without Thrombocytopenia During the ICU Stay. Supplementary Table S3. Pairwise Comparison of Admission STP (Admission PC 150–199 × 10^9^/L) Versus Admission PC ≥ 200 × 10^9^/L.

## Abbreviations

The following abbreviations are used in this manuscript:

ALI	Acute Liver Injury
aHR	adjusted Hazard Ratio
ALF	Acute Liver Failure
AKI	Acute Kidney Injury
APACHE II	Acute Physiology and Chronic Health Evaluation II
CBC	Complete Blood Count
CI	Confidence Interval
CVC	Central Venous Catheter
DIC	Disseminated Intravascular Coagulation
FFP	Fresh Frozen Plasma
GCS	Glasgow Coma Scale
HIT	Heparin-induced Thrombocytopenia
HR	Hazard Ratio
ICU	Intensive Care Unit
ICU-TP	ICU-acquired thrombocytopenia
ISTH	International Society of Thrombosis and Haemostasis
LMWH	Low Molecular Weight Heparin
MV	Mechanical Ventilation
OR	Odds Ratio
PC	Platelet Count (PC)
RBC	Red Blood Cell
RRT	Renal Replacement Therapy
STP	Subthreshold Thrombocytopenia
SOFA	Sequential Organ Failure Assessment
TP	Thrombocytopenia
VP	Vasopressor
WBC	White Blood Cell
