# Supplementary material for: Associations of Thrombocytopenia and Subthreshold Platelet Counts with Clinical Outcomes in Intensive Care Patients: A Prospective Cohort Study
Source: J Clin Med. 2026 Oct 12;15(20):7869. doi: 10.3390/jcm15207869 (PMC13705574; doi:10.3390/jcm15207869)
Supplement: Supplementary file 1 [file jcm-15-07869-s001.zip › jcm-4485924-supplementary.pdf]

Supplementary Table S1. Association Between Thrombocytopenia Severity Based on the Nadir ICU Platelet Count and Clinical Outcomes

Mortality n (%)	Mild<br>33 (28)	Moderate<br>44 (37.3)	Severe<br>19 (16.1)	Very Severe<br>22 (18.6)	p
**ICU n (%)**	**9 (27.3%; CI 15.1–44.2)**	**18 (40.9%; 27.7–55.6)**	**16 (84.2%; 62.4–94.5)**	**20 (90.9%; 72.2–97.5)**	**<0.001**
**Hospital n (%)**	**11 (33.3%; 19.8–50.4)**	**23 (52.3%; 37.9–66.2)**	**16 (84.2%; 62.4–94.5)**	**20 (90.9%; 72.2–97.5)**	**<0.001**
**28 days n (%)**	**9 (27.3%; 15.1–44.2)**	**20 (45.5%; 31.7–59.9)**	**13 (68.4%; 46.0–84.6)**	**15 (68.2%; 47.3–83.6)**	**0.006**
**90 days n (%)**	**12 (36.4%; 22.2–53.4)**	**28 (63.6%; 48.9–76.2)**	**15 (78.9%; 56.7–91.5)**	**20 (90.9%; 72.2–97.5)**	**<0.001**

Abbreviations: LOS, length of stay; ICU, Intensive Care Unit; TP, Thrombocytopenia

Thrombocytopenia severity was classified according to the lowest platelet count recorded during the ICU stay (nadir platelet count): mild, 100–149 $\times 10^9$/L; moderate, 50–99 $\times 10^9$/L; severe, 20–49 $\times 10^9$/L; and very severe, <20 $\times 10^9$/L.

Mortality estimates are presented as proportions with Wilson 95% confidence intervals.

Supplementary Table S2. Comparison of Interventions, Complications, and Outcomes Between Patients With and Without Thrombocytopenia During the ICU Stay

Variable	TP during ICU stay n=118	No TP during ICU stay n=162	p
**Interventions and organ support during the ICU stay**			
Platelet transfusion, yes n (%)	**29 (24.6)**	**3 (1.9)**	**<0.001**
Platelet count immediately before the first platelet transfusion (×109/L)*	**15 (6.5-31.5)**	**155 (150-160)**	**<0.001**
RBC Transfusion n (%)	**59 (50)**	**44 (27)**	**<0.001**
FFP/Cryoprecipitate transfusion n (%)	**45 (38.1)**	**15 (9.3)**	**<0.001**
RRT requirement, yes n (%)	**59 (50)**	**44 (27)**	**<0.001**
MV requirement, yes n (%)	**65 (55)**	**36 (22)**	**<0.001**
Vasopressor requirement, yes n (%)	**69 (58.5)**	**39 (24.1)**	**<0.001**
Thromboprophylaxis n (%)			
Heparin	1 (0.8)	1 (0.6)	1.000
LMWH	96 (81)	137 (84.6)	0.774
Other	12 (10.2)	14 (8.6)	0.681
Total	109 (92.4)	152 (93.8)	0.633
Antibiotic therapy, yes n (%)	111 (94)	143 (88)	0.14
Steroid therapy, yes n (%)	**73 (61.9)**	**67 (41.4)**	**0.001**
CVC placement, yes n (%)	**69 (58.5)**	**55 (34)**	**<0.001**
**Complications recorded during the ICU stay**			
Sepsis yes n (%)	**87 (73.7)**	**78 (48)**	**<0.001**
Hemorrhage n (%)	24 (21.2)	27 (16.7)	0.354
Grade 1-2	14 (11.9)	17 (10.5)	
Grade 3	7 (5.9)	5 (3.1)	
Grade 4	4 (3.4)	5 (3.1)	
Thrombosis, yes n (%)	4 (3.4)	9 (5.6)	0.567
DIC, yes n (%)	**40 (34)**	**9 (5.6)**	**<0.001**
AKI, yes n (%)	**65 (55)**	**60 (37)**	**0.003**
Acute Liver Injury, yes n (%)	**67 (56.8)**	**58 (35.8)**	**<0.001**
Acute Liver Failure, yes n (%)	4 (3.4)	3 (1.9)	0.46

Abbreviations: AKI, Acute Kidney Injury; CVC, Central Venous Catheter; DIC, Disseminated Intravascular Coagulation; FFP, Fresh Frozen Plasma; LMWH, Low Molecular Weight Heparin; MV, Mechanical Ventilation; RBC, Red Blood Cell; RRT, Renal Replacement Therapy; TP, Thrombocytopenia.

*The platelet count immediately before the first platelet transfusion is reported only for transfused patients (AT, n=29; NT, n=3) and represents an observed pre-transfusion value rather than a protocol-defined transfusion threshold. In the three transfused patients in the NT group, platelet transfusion was administered because of medication-associated platelet dysfunction and clinically significant bleeding despite platelet counts remaining ≥150 $\times 10^9$/L.

p values are unadjusted and relate to exploratory comparisons; they should be interpreted with caution.

Variable	PC ≥200 ×10⁹/L (n=160)	Admission STP (n=58)	Risk difference* (95% CI)	OR (95% CI)†	p‡	Holm-adjusted p§
**Admission characteristics**						
Age, years, median (IQR)	71.5 (57.2–81.7)	74.5 (63–85.2)	-	–	0.152	1.000
Female sex, n (%)	68 (42.5)	28 (48.3)	5.8 (−8.8 to 20.4)	1.26 (0.69–2.31)	0.448	1.000
GCS, median (IQR)	13 (10–15)	14 (12–15)	-	–	0.511	1.000
APACHE II, median (IQR)	20.5 (15–29)	19.5 (15–26)	-	–	0.546	1.000
SOFA, median (IQR)	5 (3–7)	4 (2–6.5)	-	–	0.344	1.000
**Interventions during ICU stay**						
Platelet transfusion, n (%)	9 (5.6)	3 (5.2)	−0.5 (−6.3 to 8.9)	0.92 (0.24–3.50)	1.000¶	1.000
RBC transfusion, n (%)	55 (34.4)	17 (29.3)	−5.1 (−17.8 to 9.4)	0.79 (0.41–1.52)	0.482	1.000
FFP/cryoprecipitate transfusion, n (%)	28 (17.5)	9 (15.5)	−2.0 (−11.7 to 10.5)	0.87 (0.38–1.96)	0.730	1.000
Invasive MV, n (%)	54 (33.8)	13 (22.4)	−11.3 (−23.0 to 2.7)	0.57 (0.28–1.14)	0.109	1.000
Vasopressor use, n (%)	55 (34.4)	14 (24.1)	−10.2 (−22.2 to 4.0)	0.61 (0.31–1.20)	0.151	1.000
**Complications during ICU stay**						
DIC, n (%)	16 (10.0)	9 (15.5)	5.5 (−3.6 to 17.5)	1.65 (0.69–3.98)	0.259	1.000
AKI, n (%)	70 (43.8)	20 (34.5)	−9.3 (−22.7 to 5.6)	0.68 (0.36–1.26)	0.219	1.000
**Clinical outcomes**						
ICU mortality, n (%)	50 (31.3)	12 (20.7)	−10.6 (−21.9 to 3.2)	0.57 (0.28–1.18)	0.127	1.000
Hospital mortality, n (%)	58 (36.3)	13 (22.4)	−13.8 (−25.5 to 0.3)	0.51 (0.25–1.02)	0.054	0.649
28-day mortality, n (%)	50 (31.3)	11 (19.0)	−12.3 (−23.3 to 1.3)	0.51 (0.25–1.08)	0.074	0.816
90-day mortality, n (%)	63 (39.4)	16 (27.6)	−11.8 (−24.3 to 2.8)	0.59 (0.30–1.13)	0.110	1.000

Supplementary Table S3. Pairwise Comparison of Admission STP (Admission PC 150–199 ×10⁹/L) Versus Admission PC ≥200 ×10⁹/L

Abbreviations: AKI, acute kidney injury; APACHE II, Acute Physiology and Chronic Health Evaluation II; CI, confidence interval; DIC, disseminated intravascular coagulation; FFP, fresh frozen plasma; GCS, Glasgow Coma Scale; ICU, intensive care unit; IQR, interquartile range; MV, mechanical ventilation; OR, odds ratio; PC, platelet count; RBC, red blood cell; SOFA, Sequential Organ Failure Assessment; STP, subthreshold thrombocytopenia.

*Risk difference (admission STP minus PC ≥200 ×10⁹/L) in percentage points, with Newcombe 95% CI; calculated for categorical variables only.
†Odds ratio for admission STP relative to PC ≥200 ×10⁹/L (reference), with Woolf 95% CI.
‡Unadjusted two-sided p-values from Pearson's chi-square test (Fisher's exact test where an expected count was <5) or the Mann–Whitney U test.
§Holm-adjusted p-values across all 16 pairwise comparisons in this table.
¶Fisher's exact test.
Classification was based on the first PC at ICU admission. These exploratory comparisons do not test equivalence; a non-significant result should not be interpreted as clinical equivalence.
